# Phosphotyrosine profiling of human cerebrospinal fluid

**DOI:** 10.1186/s12014-018-9205-1

**Published:** 2018-09-12

**Authors:** Gajanan Sathe, Chan Hyun Na, Santosh Renuse, Anil Madugundu, Marilyn Albert, Abhay Moghekar, Akhilesh Pandey

**Affiliations:** 1 Center for Molecular Medicine, National Institute of Mental Health and Neurosciences (NIMHANS), Hosur Road, Bangalore, 560029 India; 2 Institute of Bioinformatics, International Technology Park, Bangalore, 560 066 India; 3 McKusick-Nathans Institute of Genetic Medicine, Johns Hopkins University School of Medicine, Baltimore, MD 21205 USA; 4 Department of Neurology, Johns Hopkins University School of Medicine, Baltimore, MD 21205 USA; 5 Departments of Biological Chemistry, Pathology and Oncology, Johns Hopkins University School of Medicine, Baltimore, MD 21205 USA; 6 Institute for Cell Engineering, Johns Hopkins University School of Medicine, Baltimore, MD 21205 USA; 7 Manipal Academy of Higher Education (MAHE), Manipal, Karnataka 576104 India

**Keywords:** CSF, Phosphotyrosine, Proteome

## Abstract

**Background:**

Cerebrospinal fluid (CSF) is an important source of potential biomarkers that affect the brain. Biomarkers for neurodegenerative disorders are needed to assist in diagnosis, monitoring disease progression and evaluating efficacy of therapies. Recent studies have demonstrated the involvement of tyrosine kinases in neuronal cell death. Thus, neurodegeneration in the brain is related to altered tyrosine phosphorylation of proteins in the brain and identification of abnormally phosphorylated tyrosine peptides in CSF has the potential to ascertain candidate biomarkers for neurodegenerative disorders.

**Methods:**

In this study, we used an antibody-based tyrosine phosphopeptide enrichment method coupled with high resolution Orbitrap Fusion Tribrid Lumos Fourier transform mass spectrometer to catalog tyrosine phosphorylated peptides from cerebrospinal fluid. The subset of identified tyrosine phosphorylated peptides was also validated using parallel reaction monitoring (PRM)-based targeted approach.

**Results:**

To date, there are no published studies on global profiling of phosphotyrosine modifications of CSF proteins. We carried out phosphotyrosine profiling of CSF using an anti-phosphotyrosine antibody-based enrichment and analysis using high resolution Orbitrap Fusion Lumos mass spectrometer. We identified 111 phosphotyrosine peptides mapping to 66 proteins, which included 24 proteins which have not been identified in CSF previously. We then validated a set of 5 tyrosine phosphorylated peptides in an independent set of CSF samples from cognitively normal subjects, using a PRM-based targeted approach.

**Conclusions:**

The findings from this deep phosphotyrosine profiling of CSF samples have the potential to identify novel disease-related phosphotyrosine-containing peptides in CSF.

**Electronic supplementary material:**

The online version of this article (10.1186/s12014-018-9205-1) contains supplementary material, which is available to authorized users.

## Background

Cerebrospinal fluid (CSF) is a colorless, clear liquid that surrounds the brain and spinal cord [1] and conventionally acts as a shock absorber in the central nervous system by maintaining electrolytes as well as acid–base balance [2]. It also plays a crucial role in neurodevelopment and waste clearance in addition. CSF is produced from arterial blood by the choroid plexuses of the lateral and fourth ventricles. It also secretes nutrients, filters metabolites from the blood and eliminates waste products from the brain [3]. CSF regulates neuronal activity through neuropeptides and hormones [4]. As a proximal fluid, CSF reflects the health/disease state of the nervous system and is likely to have a higher concentration of protein biomarkers, which provide a direct readout of the state of the central nervous system [5]. Investigating the proteomic profile of this fluid can be beneficial in diagnosing various neurological disorders including Alzheimer’s, Parkinson’s diseases and multiple sclerosis.

Phosphorylation is one of the most commonly studied post-translational modifications (PTMs), and phosphorylation of proteins at specific sites are already used as biomarkers in neurological disorders (e.g.: phosphorylated tau in AD) [6–8]. Additionally, phosphorylation plays important roles in cell communication, signaling, aging, and cell adhesion [9, 10]. For example, phosphorylation of tyrosine residues by tyrosine kinases such as c-Abl has been reported to have a central role in neurodegeneration [11]. The biofluid phosphoproteome contains phosphorylated proteins either secreted from cells or leak from the intracellular content of damaged cells. Recent studies have shown that proteins may undergo phosphorylation in the extracellular compartment through the action of extracellular kinases [12, 13]. The discovery of novel secreted kinase VLK and Casein kinase, which are responsible for the phosphorylation of the major class of secreted proteins, has led to an increased interest in secreted phosphoproteins and their roles [14, 15]. Unfortunately, there are limited studies on direct analysis of phosphorylation in biofluids such as plasma phosphoproteins [16], CSF [17] and saliva [18]. Although several studies have been carried out on the proteomic profiling of CSF, limited studies were focused on its phosproteome [19–21]. Earlier targeted studies on neurological disorders focused on phosphorylation alteration on serine and threonine sites, including an increase in tau, synuclein phosphorylation in neurological disorders [22, 23]. Heegaard et al. in a previous study carried out phosphoproteomic profiling of cerebrospinal fluid using TiO_2_ based enrichment. They identified 56 novel phosphorylation sites on 38 proteins [19]. In another study Nakamura et al. used titanium based and IMAC enrichment followed by use of EDTA for phosphopeptides analysis leading to identification of 123 phosphopeptides [20]. Molecularly imprinted polymers that have binding affinity sites for pS or pY were also used for enrichment of phosphopeptides from CSF. This lead to identification of 47 phosphopeptides corresponding to 24 proteins [21]. All earlier mass spectrometry studies were focused on global phosphorylation of CSF.

Stoichiometrically, tyrosine phosphorylation is less abundant, and its detection is more difficult in global profiling although it plays an important role in regulation of neuronal maturation and synaptic plasticity [24]. Tyrosine phosphopeptides can be enriched using an antibody-based immunoprecipitation approach [25]. In an earlier study by *Yuan X* et al., phosphotyrosyl-proteins in CSF were detected using 2D gel electrophoresis. They identified four tyrosine phosphorylated proteins including kallikrein-6 precursor, complement C4 gamma-chain, gelsolin, and ceruloplasmin precursor. The major limitation of this approach was that the technology used was less sensitive and low throughput [26]. In this study, we carried out phosphotyrosine profiling to investigate the tyrosine phosphorylation of proteins that are detectable in the CSF. Phosphotyrosine-containing peptides were enriched by an anti-phosphotyrosine antibody-based enrichment method followed by high-resolution mass spectrometry.

## Methods

### Materials

Anti-phosphotyrosine rabbit monoclonal antibody (P-Tyr-1000) beads were obtained from Cell Signaling Technology (Danvers, MA). TPCK-treated trypsin was obtained from Worthington Biochemical Corp. (Lakewood, NJ). All other reagents used in this study were from Fisher Scientific (Pittsburgh, PA).

### Lumbar CSF samples

The sample group consisted of subjects with normal pressure hydrocephalus (NPH) and cognitively normal individuals seen as part of the BIOCARD study [27]. The participants underwent a lumbar puncture in the fasted state in the morning and the CSF was collected in polypropylene vials and stored at − 80 °C. NPH CSF was collected via an indwelling lumbar catheter and in cognitively normal individuals by lumbar puncture. We have collected CSF samples from 3 NPH and 8 cognitively normal individuals. All individuals selected for inclusion within the study provided informed consented prior to sample collection.

### Protein concentration and in-solution trypsin digestion of proteins from CSF

CSF samples were adjusted to 20 mM HEPES, 1 mM sodium orthovanadate, 2.5 mM sodium pyrophosphate, 1 mM β-glycerophosphate. CSF was concentrated 10 times by volume using 3 kDa cutoff spin columns (Microcon Ultracel YM-3, Millipore). The concentration of protein from these samples was measured using the BCA protein estimation method. We used protein from 100 ml CSF from all three samples for the in-solution digestion and the amount of the proteins present in the samples was respectively 49, 51 and 52 mg. For reduction, dithiothreitol (Sigma) was added to a final concentration of 10 mM and the mixture was incubated at 56 °C for 45 min. For alkylation, the mixture was cooled to room temperature and iodoacetamide was added to a final concentration of 20 mM and incubated at room temperature for 45 min in the dark. CSF samples were subjected to digestion with TPCK treated trypsin with 1:50 trypsin to CSF protein ratio (Worthington Biochemical Corp, Lakewood, NJ) for 12–16 h at room temperature. Protein digests were acidified by 1% trifluoroacetic acid (TFA), centrifuged at 12,000×*g* for 5 min and supernatant desalted using C_18_ Sep-Pak cartridge (Waters, Cat#WAT051910) and lyophilized.

### Immunoaffinity purification of tyrosine phosphopeptides

The lyophilized peptide mixtures were dissolved in in IAP buffer containing 50 mM MOPS pH 7.2, 10 mM sodium phosphate and 50 mM NaCl. Prior to phosphotyrosine enrichment, the P-Tyr-1000 beads (Cell Signaling Technology, Danvers, MA) were washed twice with IAP buffer at 4 °C. The peptide mixtures were then incubated with P-Tyr-1000 beads for 30 min with gentle rotation. To remove non-specifically bound peptides, the beads were washed thrice with ice cold IAP buffer and twice with ice cold water. Elution of enriched peptides from beads was carried out at room temperature using 0.15% TFA. This step was repeated twice and followed by cleanup of the samples using C_18_ Stage tips.

### LC–MS/MS analysis of enriched peptides

The enriched phosphotyrosine-containing peptides were analyzed on an Orbitrap Fusion Lumos Tribrid mass spectrometer (Thermo Electron, Bremen, Germany) interfaced with EASY-nLC II nanoflow liquid chromatography system equipped with an EASY-Spray ion source. (Thermo Scientific, Odense, Denmark). Peptide digests were reconstituted in 0.1% formic acid and loaded onto trap column (Thermo Scientific™ Acclaim™ PepMap™ 100 C18 LC) at a flow rate of 3 µl/min. Peptides were separated on an analytical column (EASY-Spray™ LC) at a flow rate of 300 nl/min using a step gradient of 5–18% solvent B (0.1% formic acid in 95% acetonitrile) for first 110 min and 18–30% solvent B for 110–160 min. The total run time was set to 180 min. The mass spectrometer was operated in a data-dependent acquisition mode. A survey full scan MS (from *m/z* 350–1500) was acquired in the Orbitrap with a resolution of 120,000 at 400 *m/z*. The AGC target for MS1 was set as 1 × 10^6^ and ion filling time set 60 ms. The most intense ions with charge state ≥ 2 were isolated in 3 s cycle and fragmented using HCD fragmentation with 32% normalized collision energy and detected at a mass resolution of 30,000 at 200 *m/z*. The AGC target for MS/MS was set as 5 × 10^4^ and ion filling time set 200 ms dynamic exclusion was set for 30 s with a 10 ppm mass window.

For the PRM analysis, the enriched phosphotyrosine containing peptides were analyzed on an Orbitrap Fusion Tribrid mass spectrometer interfaced with EASY-nLC II nanoflow liquid chromatography system. Peptide digests were reconstituted in 0.1% formic acid and loaded onto a trap column at a flow rate of 3 µl/min and resolved on analytical column. The mass spectrometer was operated in data-independent acquisition PRM mode. A survey full scan MS (from m/z 350–1700) was acquired in the Orbitrap at a resolution of 120,000 at 400 *m/z*. A targeted list of precursor ions with charge state ≥ 2 were isolated and fragmented using HCD fragmentation with 32% normalized collision and detected at a mass resolution of 30,000 at 400 *m/z*. The data were subsequently analyzed using Skyline [28].

### Data analysis

The MS/MS database searches were carried out using SEQUEST search algorithms against RefSeq human protein database using Proteome Discoverer 2.1 (Thermo Fisher Scientific, Bremen, Germany). The workflow included spectrum selector, SEQUEST search nodes, peptide validator and phosphoRS nodes. Oxidation of methionine, phosphorylation at serine, threonine and tyrosine (+ 79.966 Da) were set as variable modifications and carbamidomethylation of cysteine was set as a fixed modification. MS and MS/MS mass tolerances were set to 10 ppm and 0.05 Da, respectively. Trypsin was specified as protease and a maximum of one missed cleavage was allowed. Target-decoy database searches used for calculation of false discovery rate (FDR) and for peptide identification FDR was set at 1%. The probability of the phosphorylation site was calculated using phosphoRS 3.1 node in the Proteome Discoverer. Phosphopeptides with > 75% localization probability was considered for further analysis.

### Availability of data

The mass spectrometry derived data have been deposited to the ProteomeXchange Consortium (http://proteomecentral.proteomexchange.org) via the PRIDE partner repository with the dataset identifierPXD009152.

### Bioinformatics analysis

Molecular function and localization of phosphoproteins was obtained from the Human Protein Reference Database (HPRD) [29]. The involvement of theses phosphoproteins in biological processes was also obtained from HPRD.

## Results

### LC–MS/MS analysis of tyrosine phosphorylated peptides in CSF

**Fig. 1:**
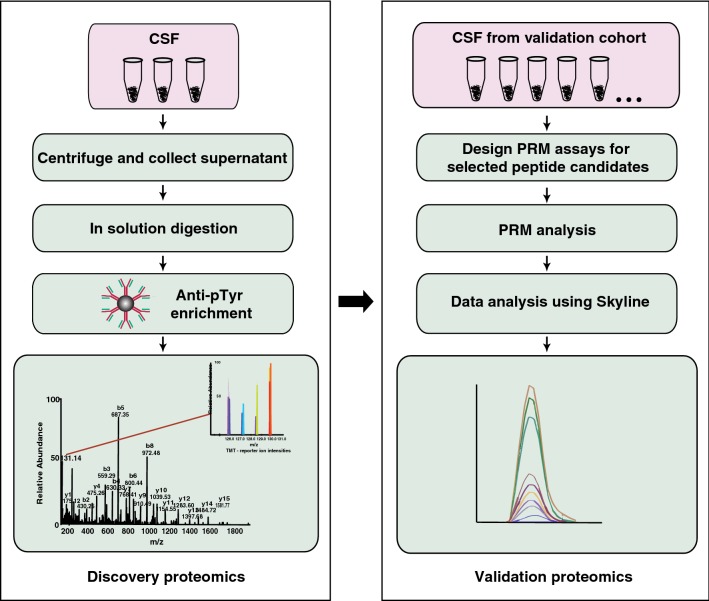
A schematic of the workflow used to study the phosphotyrosine profiling of CSF. Phosphotyrosine peptides from CSF of three NPH patients were enriched using antibody-based approach and enriched peptides were analyzed on mass spectrometer. The subset of phosphotyrosine sites identified in our discovery experiment were validated in the another set of 8 normal individual CSF using parallel reaction monitoring (PRM) assays

**Fig. 2:**
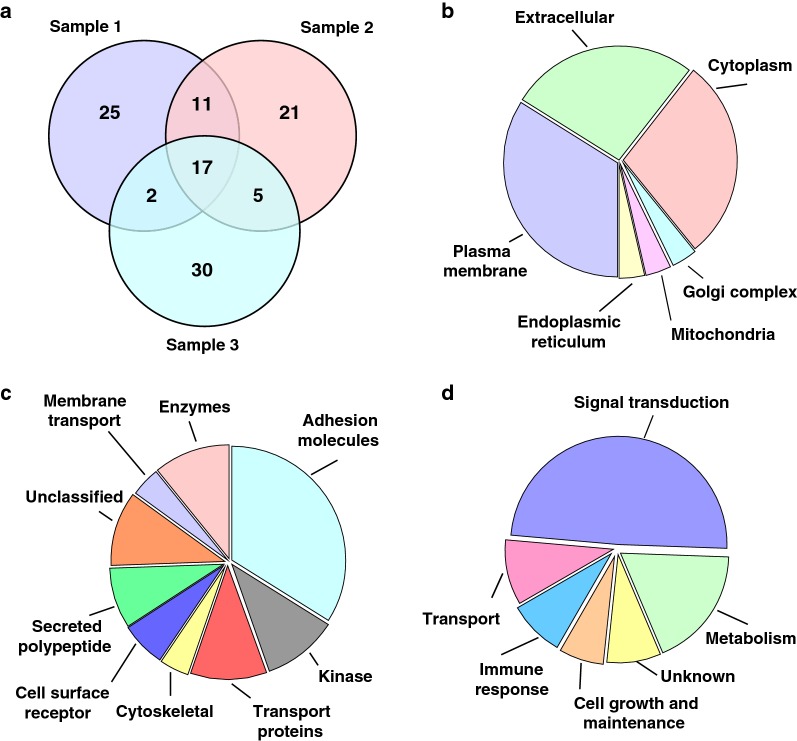
Summary of phosphotyrosine-containing peptides/proteins in CSF. **a** Overlap of phosphotyrosine peptides identified from three individuals. We identified 18 phosphopeptides common across the CSF of three individuals, **b** localization of tyrosine phosphorylated proteins identified in CSF of NPH patients. **c** Functional categorization of tyrosine phosphorylated protein identified in CSF based on the molecular function, **d** functional categorization of tyrosine phosphorylated protein identified in CSF based on the biological processes

The CSF proteins were digested with trypsin, phosphotyrosine peptides were enriched, and LC–MS/MS analysis was conducted as described in Methods [25, 30, 31] (Fig. 1). We identified 111 phosphotyrosine peptides mapping to 66 proteins (Additional file 1: Table S1) of which 38 were identified from at least two samples (Fig. 2a). These identified phosphotyrosine peptides are corresponding to 66 proteins (Additional file 2: Table S2). We have used tyrosine phosphorylated peptide immunoaffinity enrichment-based technique along with high-resolution mass spectrometry on an Orbitrap Fusion Lumos Tribrid mass spectrometer—the latest generation mass spectrometer for deep characterization of phosphotyrosine peptides in CSF.

### Classification of tyrosine phosphorylated CSF proteins using gene ontology

To obtain deeper biological insights into the identified CSF tyrosine phosphorylated proteins, we categorized them based on their subcellular localization, molecular function and biological process (Fig. 2b–d). The secretory proteins were mostly hormones, cytokines and proteases that possess important regulatory functions and are released for cell to cell interaction and communication. Of the 66 proteins identified, 33 proteins have signal peptides and 13 have transmembrane domains. This analysis reveals that the majority of tyrosine phosphorylated proteins in CSF are destined towards the secretory pathway. Our analysis showed that a majority of the tyrosine phosphorylated proteins were localized to the plasma membrane (34%), followed by extracellular matrix (26%), cytosol (26%) and endoplasmic reticulum (4%) (Fig. 2b). Most of the proteins were found to be involved in cell adhesion activity, transport activity or kinase activity (Fig. 2c). A majority of the proteins identified were cell adhesion proteins, which included CHL1, SPP1 and NRXN3. The CHL1 cell adhesion molecule L1 like is a neural cell adhesion molecules and it is significantly associated with schizophrenia [32, 33]. SPP1 (secreted phosphoprotein 1) and NRXN3 (neurexin 3) are already known to be associated with Alzheimer’s disease [34, 35]. The other major categories of proteins identified were kinases (15%), cytoskeletal proteins (14%), membrane and cell junction proteins (13%) and the transport/carrier proteins. The identified CSF proteins were also categorized based on their biological processes. Most of these proteins were found to be involved in cell communication and signal transduction, followed by transport, cell growth and energy pathways as shown in Fig. 2d.

### Targeted analysis of a subset of tyrosine phosphorylated peptides in CSF

The results from the discovery experiments were subsequently validated in CSF of cognitively normal individuals (N = 8) using a targeted parallel reaction monitoring (PRM) approach. We selected a set of tyrosine phosphorylated peptides based on their abundance and biological role for analysis in PRM mode. The tyrosine phosphorylated peptides identified in normal individual CSF using PRM mode includes proteins DBI (diazepam binding inhibitor), acyl-CoA binding protein, B4GAT1 (beta-1, 3-N-acetylglucosaminyltransferase), CD59, transferrin and Thy-1 cell surface antigen. The list of the tyrosine phosphorylated peptides and its corresponding proteins are shown in Table 1. The relative abundance of the each of the phosphopeptides in the different samples is shown in Additional file 3: Figure S1.

**Table 1:** A list of proteins that are validated using PRM

Gene symbol	Protein	Peptide sequence	Site
*DBI*	Diazepam binding inhibitor, acyl-CoA binding protein	TKPSDEEMLFIYGHYK	Y90
*TF*	Transferrin	EGYYGYTGAFR	Y536
*B4GAT1*	Beta-1, 4-glucuronyltransferase 1	YEAAVPDPR	Y163
*CD59*	CD59 glycoprotein preproprotein	ENELTYYCCK	Y86
*THY*-*1*	Thy-1 cell surface antigen	VLYLSAFTSK	Y90

## Discussion

### Phosphotyrosine profiling of cerebrospinal fluid

Since the alterations in tyrosine phosphorylation of proteins in the brain can be reflected in the CSF, such proteins can be excellent candidates as biomarkers for neurodegenerative diseases such as AD and PD. In spite of this, there are no reports that describe phosphotyrosine profiling of proteins in CSF. Proteins can be phosphorylated on serine, threonine and tyrosine. Of these, the phosphorylation on tyrosine residues is much less abundant than either serine or threonine residues. Owing to this, the identification of phosphotyrosine modifications is mostly conducted after enriching phosphotyrosine-containing peptides using anti-phosphotyrosine antibodies from large amounts of proteins (> 20 mg). Because the protein concentration of CSF is only ~ 0.15 to 0.6 mg/ml, large volumes of CSF are required for such an analysis. To overcome this limitation, we used three sets of CSF samples (100 ml each generating ~ 50 mg protein in each instance) obtained from subjects with NPH and processed them independently. In this study, we employed antibody-based enrichment with Orbitrap Fusion Lumos Tribrid mass spectrometer—the latest generation mass spectrometer for identification of tyrosine phosphorylation in the cerebrospinal fluid. We identified 111 phosphotyrosine peptides corresponds to 66 proteins.

### Proteins uniquely identified in this study

To identify proteins which have not been previously reported in the CSF, we compared our data with proteins cataloged in the CSF-PR database. CSF-PR is an online repository of mass spectrometry-based proteomics experiments on human cerebrospinal fluid [36]. We identified 24 proteins that have not been previously detected in cerebrospinal fluid. A partial list of uniquely identified proteins in this study is provided in Table 2. This list includes proteins CWF19 like 1, cell cycle control (CWF19L1), inositol polyphosphate-5-phosphatase D (INPP5D), beta-1, 4-glucuronyltransferase 1 (B4GAT1), protein kinase C delta (PRKCD), NCK adaptor protein 2 (NCK2), glucose-6-phosphate dehydrogenase (G6PD).

**Table 2:** A list of proteins identified that are unique in this study

Gene symbol	Protein	Peptide sequence	Site
*CWF19L1*	CWF19 like 1, cell cycle control	CGSALVSSLATGLKPRYHFAALEK	Y192
*INPP5D*	Inositol polyphosphate-5-phosphatase D	EKLYDFVK	Y865
*B4GAT1*	Beta-1, 4-glucuronyltransferase 1	YEAAVPDPR	Y163
*PTPN11*	Tyrosine-protein phosphatase non-receptor type 11	IQNTGDYYDLYGGEK	Y584
*G6PD*	Glucose-6-phosphate dehydrogenase	VQPNEAVYTK	Y432
*FGR*	FGR proto-oncogene	LIKDDEYNPCQGSK	Y412
*PXN*	Paxillin	VGEEEHVYSFPNK	Y124
*CALM 2*	Calmodulin 2	DGNGYISAAELR	Y148

**Fig. 3:**
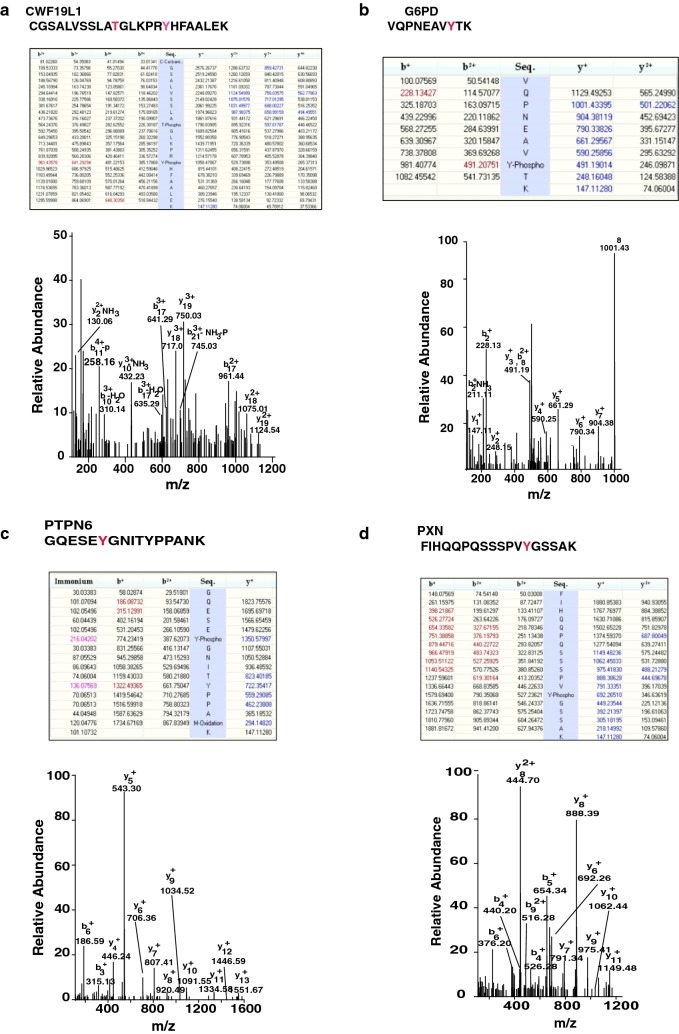
MS/MS spectra for the phosphotyrosine-containing peptides/proteins uniquely identified in ours study. **a** MS/MS spectra for doubly phosphorylated peptide identified from CWF19, **b** representative MS/MS spectra for phosphorylated peptide identified from G6PD, **c** representative MS/MS spectra for phosphorylated peptide identified from PTPN6 and **d** representative MS/MS spectra for phosphorylated peptide identified from PXN

Mutations in the CWF19 gene, which encodes CWF19 protein family have been associated with ataxia and mild mental retardation [37]. CWF19 has a strong association with late-onset Alzheimer’s disease [38]. Our study identified phosphorylation of CWF19 at Y192 and T186 as it was located on the same phosphopeptide. Phosphorylation of these sites has not been reported previously. The representative MS/MS for the doubly phosphorylated peptide is shown in Fig. 3a. Glucose-6-phosphate dehydrogenase is a cytosolic enzyme and its main function is to produce NADPH, an important electron donor. Activity of G6PD is increased in the inferior temporal cortex of Alzheimer individuals [39]. G6PD plays an important role in compensating oxidative stress produced in Alzheimer’s disease [40]. In another study, G6PD enzyme activity in serum of AD and control subjects was measured. G6PD activity is two times higher in AD patients as compared to controls [41]. We identified G6PD phosphorylation at Y401 in CSF. The representative MS/MS spectra is shown in the Fig. 3b.

PTPN6 belongs to a member of the protein tyrosine phosphatase (PTP) family. It involved in the cell differentiation, cell growth, differentiation, mitotic cycle, and oncogenic transformation [42]. PTPN6 expression was increased in glioma and its expression correlating with poor survival. Its expression controlled epigenetically and impacts the response to chemotherapy [43]. In our analysis, we detected phosphorylation at Y 584. The MS/MS spectrum for this peptide is shown in Fig. 3c. Paxillin (PXN) is involved in actin-membrane attachment at sites of cell adhesion to the extracellular matrix. It is an adapter protein which plays an important scaffolding role at focal adhesions by employing structural and signaling molecules involved in cell movement and migration, when phosphorylated on specific Tyr and Ser residues [44]. We have detected phosphorylated paxillin in CSF. The MS/MS of the phosphopeptide is shown in the Fig. 3d. These newly identified CSF proteins will help to increase knowledge of extracellular signaling and understanding pathology of neurological disorders.

### Targeted analysis of tyrosine phosphorylated CSF proteins

The results from the discovery experiments were subsequently validated in CSF of cognitively normal individuals using a targeted parallel reaction monitoring (PRM) approach. This tyrosine phosphorylated peptides corresponds to the proteins DBI (diazepam binding inhibitor), acyl-CoA binding protein, B4GAT1 (beta-1, 3-N-acetylglucosaminyltransferase), CD59, transferrin and Thy-1 cell surface antigen. Validation of these tyrosine phosphorylated peptides in 8 CSF samples from cognitively normal healthy controls is a preliminary finding that needs to be replicated in a larger cohort. Unlike other biofluids, CSF is difficult to obtain, especially CSF from cognitively normal healthy controls. Although the sample size for the validation is small, these findings are quite promising.

**Fig. 4:**
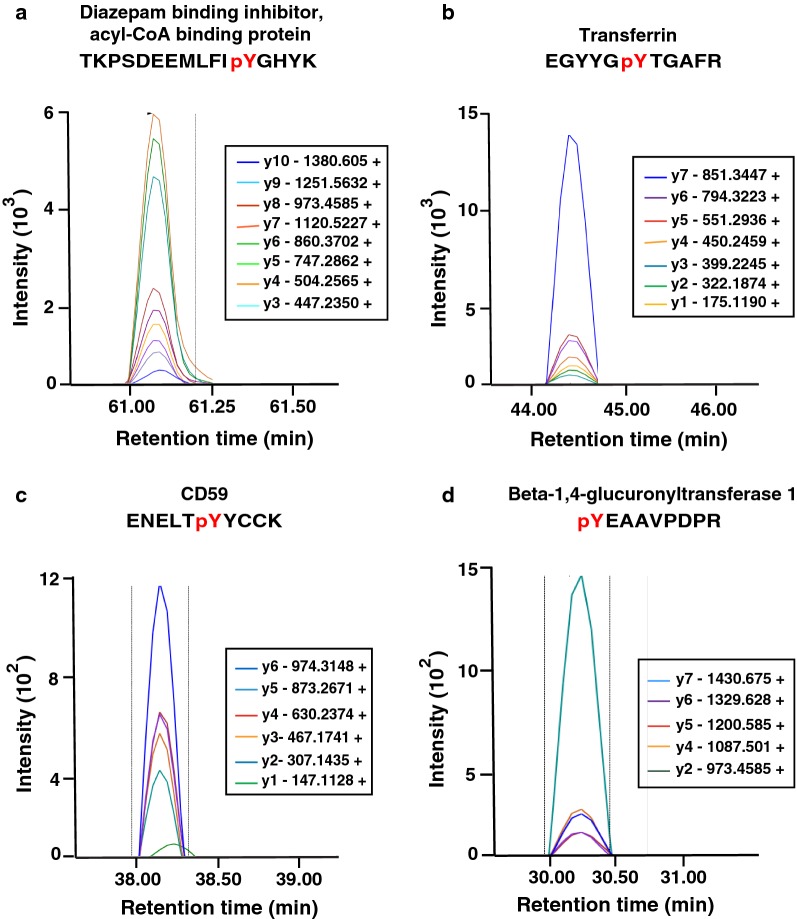
Validation of tyrosine phosphorylated peptides in control CSF samples by parallel reaction monitoring. **a** Diazepam binding inhibitor, acyl-CoA binding protein (DBI), **b** transferrin (TF) and **c** CD 59 and **d** beta-1,4-glucuronyltransferase 1(B4GAT1)

DBI (diazepam binding inhibitor) is a polypeptide that was initially identified from rat brains [45]. This protein is involved in lipid metabolism and the displacement of beta-carbolines and benzodiazepines, which regulate signal transduction at type-a gamma-aminobutyric acid receptor located in brain synapses. Multiple missense mutations have been reported in the DBI gene in schizophrenia [46]. It regulates steroidogenesis in mitochondria and also glucose-stimulated insulin secretion [47]. DBI gene polymorphisms are also associated with anxiety disorders [48]. As DBI can be a potential candidate gene for psychiatric phenotypes including anxiety, mood, and psychotic disorders, its concentration has been investigated in the plasma of epileptic patients and found to be elevated [49]. The transitions for the peptide corresponding to phosphorylated DBI are shown in Fig. 4a.

Transferrin plays important roles in iron transport from the intestine, reticuloendothelial system, and liver parenchymal cells to all cells in the body for proliferation. It also plays important roles in the removal of allergens from the serum. Several mass-spectrometry based method were established for the detection of glycoforms for diagnosis of congenital disorders [50]. Mutations in transferrin lead to an increased risk of Alzheimer’s disease [51]. The N-glycan proteoform of CSF transferrin is a potential biomarker for normal pressure hydrocephalus (iNPH) [52]. Transferrin has recently been shown to be a potent in vitro inhibitor of self-association of Aβ [53]. The transitions for the peptide corresponding to phosphorylated transferrin are shown in Fig. 4b.

CD 59 is a surface glycoprotein that controls complement-mediated cell lysis, which has been identified previously in plasma, urine, saliva and CSF [54]. It has been reported that the concentration of CD 59 is elevated in multiple sclerosis patients [55]. We have identified phosphorylation of CD 59 at Y87. The transitions for the peptide corresponding to phosphorylated CD 59 are shown in Fig. 4c. Thy-1 antigen is a cell surface glycoprotein, which is the most abundant glycoprotein on mammalian neurons [56, 57]. It has also been reported as a cancer stem cell marker in high grade gliomas [58]. In the nervous system, Thy-1 plays an important role in cell communication [59]. We have detected tyrosine phosphorylated sites on Thy-1 in CSF samples using the PRM approach. B4GAT1 is a member of the beta-1,3-N-acetylglucosaminyltransferase family and it is a transmembrane protein having enzymatic activity. A truncating mutation in B4GAT1 is known to causes severe Walker-Warburg syndrome, a congenital muscular dystrophy [60]. Dystroglycan organizes basement of cell membrane by interacting ligands in the extracellular matrix. BGAT1 play important role in activation of dystroglycan [61]. The transitions for the peptide corresponding to phosphorylated Beta-1,4-glucuronyltransferase 1 are shown in Fig. 4d. The PRM analysis of normal CSF samples confirms that these tyrosine phosphorylated peptides are easily detected in individual normal CSF samples using the PRM approach.

## Conclusions

In this study, we have investigated tyrosine phosphorylation in cerebrospinal fluid and identified several novel phosphosites. The study represents the first deep phosphotyrosine proteome profiling of cerebrospinal fluid. Further work is necessary in larger cohorts comparing cognitively normal and diseased subjects to determine if any of these phosphoproteins play a role in pathophysiology or can serve as biomarkers in neurological disorders.

## Additional files

**Additional file 1: Table S1.** A summary of tyrosine phosphopeptides identified in CSF.

**Additional file 2: Table S2.** A summary of tyrosine phosphorylated proteins identified in CSF.

**Additional file 3: Figure S1.** A relative abundance of the tyrosine phosphorylated peptides in the CSF samples.
