# Supplementary material for: Phosphotyrosine profiling of human cerebrospinal fluid
Source: Clin Proteomics. 2018 Sep 12;15:29. doi: 10.1186/s12014-018-9205-1 (PMC6136184; doi:10.1186/s12014-018-9205-1)
Supplement: Supplementary file 3 — Additional file 3: Figure S1. A relative abundance of the tyrosine phosphorylated peptides in the CSF samples. [file 12014_2018_9205_MOESM3_ESM.pdf]

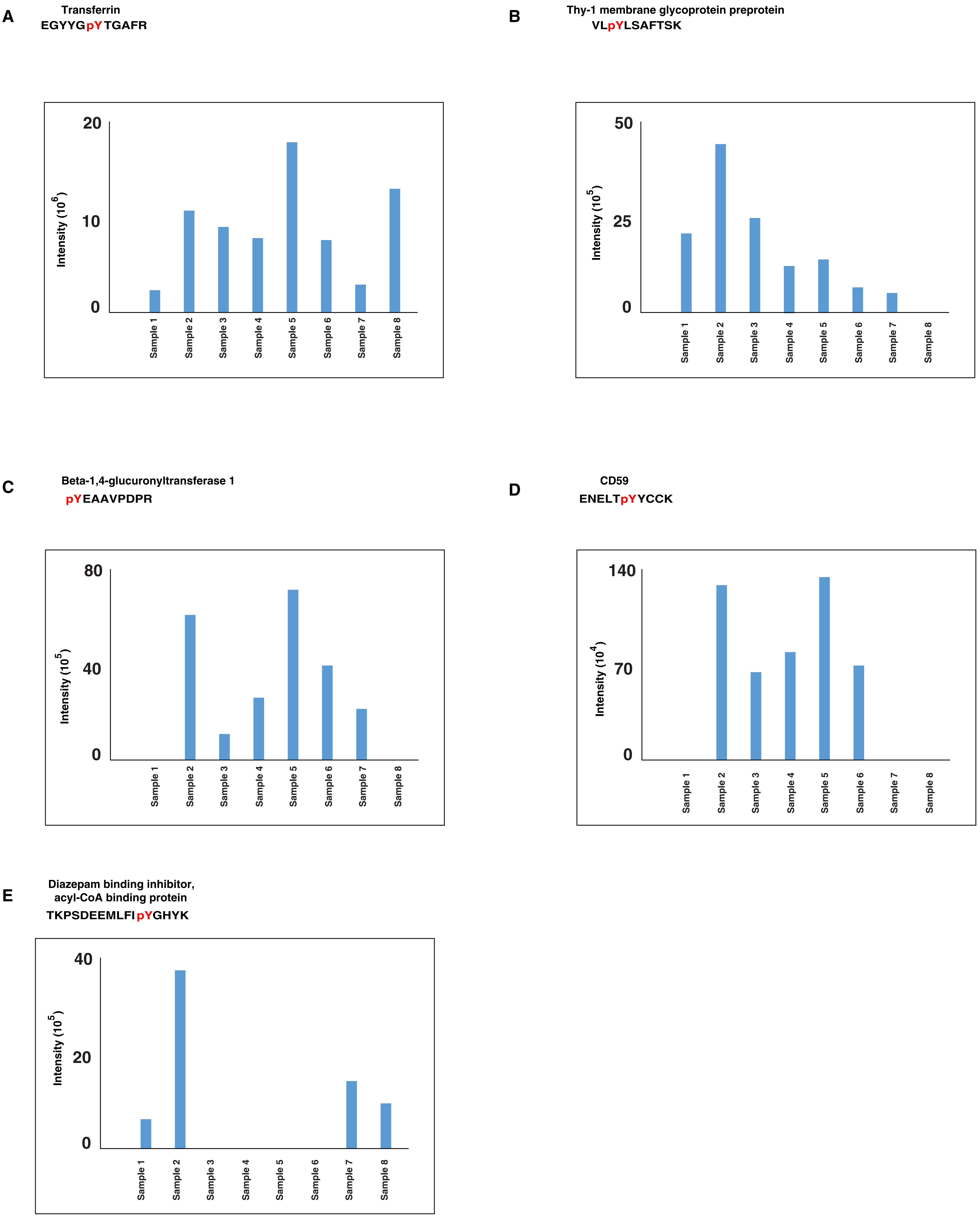
A
Transferrin
EGYYGpYTGAFR
Intensity (10^6)
20
10
0
Sample 1
Sample 2
Sample 3
Sample 4
Sample 5
Sample 6
Sample 7
Sample 8
B
Thy-1 membrane glycoprotein preprotein
VLpYLSAFTSK
Intensity (10^5)
50
25
0
Sample 1
Sample 2
Sample 3
Sample 4
Sample 5
Sample 6
Sample 7
Sample 8
C
Beta-1,4-glucuronyltransferase 1
pYEAAVPDPR
Intensity (10^5)
80
40
0
Sample 1
Sample 2
Sample 3
Sample 4
Sample 5
Sample 6
Sample 7
Sample 8
D
CD59
ENELTpYYCCK
Intensity (10^4)
140
70
0
Sample 1
Sample 2
Sample 3
Sample 4
Sample 5
Sample 6
Sample 7
Sample 8
E
Diazepam binding inhibitor,
acyl-CoA binding protein
TKPSDEEMLFIpYGHYK
Intensity (10^5)
40
20
0
Sample 1
Sample 2
Sample 3
Sample 4
Sample 5
Sample 6
Sample 7
Sample 8
